# Development and Validation of a Cross-Device Platform for Anhedonia Trend Visualization by Using Ecological Momentary Assessment and Moving Averages (Part I): Protocol for a Methodological Pilot Study

**DOI:** 10.2196/84024

**Published:** 2025-12-18

**Authors:** Yen-Chung Ho, Chia-Ling Fang, Yung-Chieh Ching, Hsiu-Ju Chang, Jiun-Yi Wang, Charles CN Wang

**Affiliations:** 1 School of Nursing College of Nursing Asia University Taichung Taiwan; 2 Department of Nursing College of Nursing National Yang Ming Chiao Tung University Taipei Taiwan; 3 Efficient Smart Care Research Center College of Nursing National Yang Ming Chiao Tung University Taipei Taiwan; 4 Department of Healthcare Administration College of Medical and Health Sciences Asia University Taichung Taiwan; 5 Department of Medical Research China Medical University Hospital China Medical University Taichung Taiwan; 6 Department of Bioinformatics and Medical Engineering Asia University Taichung Taiwan; 7 Center for Precision Health Research Asia University Taichung Taiwan

**Keywords:** anhedonia, Snaith–Hamilton pleasure scale, ecological momentary assessment, moving average, early warning systems, digital health, mHealth, data visualization

## Abstract

**Background:**

Anhedonia is a core symptom of depressive disorders. Although group-level cutoff points for the Snaith–Hamilton Pleasure Scale (SHAPS) are established, real-time, individual-level monitoring remains limited. Ecological momentary assessment (EMA) enables high-frequency sampling, and simple moving averages (SMA; 7/14/30 days) offer an interpretable way to smooth daily signals and surface early-warning trends.

**Objective:**

The aim of this study was to develop and validate a cross-device platform that collects EMA-based SHAPS responses and visualizes SMA trends for individual anhedonia monitoring. This part I protocol focuses on platform design, feasibility, usability, data quality, and initial analytical validity to inform subsequent evaluation.

**Methods:**

We will conduct a single-arm methodological pilot in approximately 24 adults, recruited from an outpatient psychiatry clinic (clinical cohort) and a university setting (nonclinical cohort). After baseline assessment, participants will complete 30 days of daily EMA-SHAPS. Primary outcomes are feasibility (adherence, missingness patterns, timing fidelity) and usability/face validity (eg, System Usability Scale, qualitative feedback). We will also describe expert content validity ratings for key visual elements and exploratory analytic checks of within-person variability and the descriptive behavior of 7/14/30-day moving averages; these analyses are not powered for confirmatory hypothesis testing.

**Results:**

This protocol specifies the design–validate sequence, recruitment procedures, and predefined adherence and missing-data rules for computing moving averages. Recruitment for the year-1 feasibility pilot is scheduled to begin in March 2026 following institutional review board approval (approval 114006; approval date: June 10, 2025), with feasibility metrics, usability indices, and parameter estimates used to refine alert logic and power calculations for a planned part II evaluation.

**Conclusions:**

Combining EMA-SHAPS with SMA-based trend visualizations may provide an interpretable way to summarize daily anhedonia signals and generate candidate early-warning indicators. This methodological pilot will deliver feasibility, usability, and preliminary analytic parameters needed to support a subsequent controlled evaluation of the platform’s clinical utility.

**International Registered Report Identifier (IRRID):**

PRR1-10.2196/84024

## Introduction

### Background

Major depressive disorder is a leading cause of disability worldwide, with enduring effects on social functioning, quality of life, and health care utilization [1]. Among its heterogeneous features, anhedonia—the diminished capacity to experience pleasure—is both a core diagnostic criterion and a prognostically salient dimension linked to poorer outcomes [2,3].

The Snaith–Hamilton Pleasure Scale (SHAPS) is one of the most widely used self-report instruments for assessing hedonic tone, with robust psychometric support across clinical and nonclinical samples [4,5]. Cross-cultural work has further demonstrated utility in Chinese-speaking contexts, facilitating screening and risk stratification [6]. Building on this literature, our prior work established group-level SHAPS cutoff points to differentiate clinical from nonclinical cases, enabling population-level identification of high-risk groups [7].

However, thresholds optimized for between-person discrimination are not designed for dynamic, within-person monitoring. Traditional assessments also rely on retrospective recall, which is vulnerable to memory biases and insensitive to short-timescale fluctuations [8-10]. Ecological momentary assessment (EMA) addresses these limitations by repeatedly sampling experiences in naturalistic settings, enabling high-frequency, context-sensitive tracking of affect and behavior [9-11]. Converging evidence suggests that changes in momentary affect and variance can precede transitions in depressive states, motivating early-warning approaches that leverage intensive longitudinal data [12-14].

The analytic challenge is to convert dense EMA streams into signals that are both statistically principled and clinically interpretable. Simple moving averages (SMAs) provide an intuitive smoothing approach long established in time-series analysis [15-17] and are closely related to exponentially weighted moving averages used for sensitive change detection [18]. Short-window averages emphasize responsiveness to recent changes, whereas longer windows emphasize stability; jointly visualized, they can reveal trend direction, volatility, and potential regime shifts in a manner that is easily communicated to clinicians. In parallel domains, rule-based use of multiple moving averages has been shown to detect regime changes in complex noisy systems, underscoring the translational potential of this interpretable family of filters [19,20]. In public health surveillance, related smoothing and alignment procedures such as delay correction and multi‑indicator integration are routinely applied to stabilize noisy signals and improve interpretability [21,22].

At the delivery layer, smartphone-enabled platforms now make it feasible to deploy EMA at scale and integrate active reports with passive sensing, a paradigm often described as digital phenotyping [23,24]. For anhedonia monitoring, combining EMA-based SHAPS with SMA-based trend visualization may offer a pragmatic balance between statistical rigor and bedside interpretability: short/medium/long SMA windows (7/14/30 d) support multi-timescale inspection, while alert candidates (eg, fast–slow crossovers; deviations from individualized baselines) can be parameterized and evaluated within a transparent framework grounded in prior early-warning theory [12-14,18].

To our knowledge, this protocol is the first to systematically integrate EMA-SHAPS with SMA-based trend/alert visualization in a cross-device platform and to prospectively evaluate feasibility, usability, and initial analytical validity. Part I (this study) emphasizes platform design, human-factors evaluation, and parameter estimation to finalize alert logic (see Table 1 for feasibility of borrowing moving‑average theory for emotional monitoring).

**Table 1:** Feasibility of borrowing moving‑average theory for emotional monitoring.

Indicator/rule	How it works (moving average domain)	Mapping to psychiatry (EMA^a^‑SHAPS^b^)	Clinical interpretation and part I feasibility
Short‑window SMA^c^ (7 d)	Emphasizes most recent observations, fast response to changes, higher sensitivity to noise	Smooths day‑to‑day SHAPS to expose short‑horizon fluctuations in hedonic tone	Early movement detector; may flag brief dips/spikes. Feasible with 30‑d diary; risk of false positives mitigated by corroboration with 14/30‑d windows
Medium‑window SMA (14 d)	Balances responsiveness and stability, dampens idiosyncratic daily noise	Tracks fortnight trends in anhedonia burden, complements 7‑d and 30‑d perspectives	Context anchor for “meaningful change.” Feasible and robust in 30‑d series; supports cross‑window triangulation
Long‑window SMA (30 d)	Maximal smoothing within part I; highlights macrotrend and regime shifts	Approximates a monthly hedonic baseline for each participant	Stability reference; fewer false alarms but slower to turn. Feasible by design (30‑d pilot).
Fast–slow crossover (7 over 30)	When short SMA crosses long SMA, indicates potential regime change in level or trend.	Up‑cross: deterioration momentum in anhedonia; down‑cross: improvement momentum	Operational early‑warning candidate; parameterized and logged without triggering actions in part I

^a^EMA: ecological momentary assessment.

^b^SHAPS: Snaith–Hamilton Pleasure Scale.

^c^SMA: simple moving average.

### Objectives (Part I)

This part I study specifies development and preliminary validation objectives for a cross-device SHAPS platform with 7/14/30-day SMA trend and alert visualizations. The focus is on feasibility, usability, content validity, and exploratory analytic behavior of moving-average–based indicators, with the aim of informing a subsequent, adequately powered part II evaluation.

### Primary Objective

The primary objective is to develop a cross-device platform that (1) collects daily EMA-based SHAPS responses for 30 days, (2) computes and visualizes SMA (7/14/30 d) trends, and (3) implements candidate alert logic, including fast–slow moving-average crossovers and deviations from individualized baselines.

### Secondary Objectives

The secondary objectives are as follows:

1. Feasibility and adherence: quantify completion/adherence rates, missingness patterns, and timing fidelity of entries.
2. Usability and face validity: evaluate user experience (eg, System Usability Scale) and perceived interpretability/acceptability of trend and alert panels.
3. Expert content validity: obtain expert ratings of content relevance/clarity for key interface elements and alert explanations (eg, content validity index).
4. Analytical checks: provide descriptive summaries of internal consistency of EMA-SHAPS, within-person variability, and the behavior of 7/14/30-day SMA windows (eg, correlations between daily SHAPS and windowed averages, simple indicators of trend stability), recognizing that this pilot is not powered for robust inferential tests.
5. Exploratory objectives: Estimate plausible parameter ranges for alert logic (eg, crossover lengths, thresholds, and persistence rules) and describe how these candidate rules behave in this small pilot sample, with the sole purpose of refining design assumptions and power calculations for a planned part II evaluation against an individual-level SHAPS benchmark.

## Methods

### Study Design

This part I development and validation study follows a design‑validate sequence—needs assessment, system design and implementation, and a 30‑day pilot in clinical and nonclinical cohorts—to evaluate feasibility and validity. A month-by-month timeline of the design–validate sequence and key milestones is provided in Table 2.

**Table 2:** Part I study timeline and milestones (August 2025 to July 2026).

Activities	August 2025	September	October	November	December	January 2026	February	March	April	May	June	July 2026
Requirements analysis and system architecture design	✓	✓	✓	✓	✓	✓						
Frontend/backend development and core feature implementation	✓	✓	✓	✓	✓	✓						
System testing and performance optimization				✓	✓	✓						
Platform validation: face validity and user experience testing							✓					
Platform validation: content validity							✓					
Pilot: participant recruitment and screening								✓	✓	✓		
Pilot: 30-d EMA^a^-SHAPS^b^ data collection								✓	✓	✓		
Pilot: user feedback synthesis								✓	✓	✓		
Results integration and report writing											✓	✓
Synthesis of development outputs/preparation for year 2											✓	✓

^a^EMA: ecological momentary assessment.

^b^SHAPS: Snaith–Hamilton Pleasure Scale.

### Measurements

The following measurements were performed.

#### Demographic Characteristics

The cross-device SHAPS platform will collect demographic data on gender, age, educational attainment, employment, marriage, region of residence, and socioeconomic status in both groups.

#### SHAPS

The cross-device SHAPS platform will measure anhedonia by using SHAPS, a 14-item self-report measure that captures recent hedonic experience across 4 domains: interests/pastimes, social interaction, sensory experience, and food/drink [4]. Multiple language versions are available, including simplified and traditional Chinese. The simplified Chinese version uses a 4-point Likert response format from “definitely agree” to “definitely disagree,” yielding total scores from 14 to 56, with reported test-retest reliability of 0.64 and Cronbach α of 0.85 [6]. The traditional Chinese version has demonstrated good readability and ease of response in pilot testing (n=13), indicating favorable face validity, and has shown test–retest reliability of 0.87 with Cronbach α of 0.91. Convergent and discriminant validity are supported by positive correlations with Patient Health Questionnaire-9 items (*r*=0.52) and the Positive and Negative Suicide Ideation–Negative Suicidal Ideation subscale (*r*=0.28), and negative correlations with self-esteem (*r*=−0.51) and the Positive and Negative Suicide Ideation–Positive Ideation subscale (*r*=−0.54). Collectively, these findings indicate that the Chinese versions of the SHAPS are reliable and valid [25].

### Study Population and Setting

Participants will be recruited from two settings: (1) an outpatient psychiatry clinic at the collaborating medical center (clinical cohort) and (2) a university campus (nonclinical cohort). The clinical cohort will consist of adults aged 18-65 years with a treating psychiatrist–assigned ICD-10 (International Classification of Diseases, 10th Revision) mood disorder diagnosis in the F32-F39 range documented in the medical record (eg, depressive episode [F32.-], recurrent depressive disorder [F33.-], other persistent mood disorders [F34.-, F34.9], and unspecified mood disorder [F39.-]). The nonclinical cohort will consist of university students aged 18-23 years recruited from campus settings as an age-restricted comparison group. After baseline assessment, all participants will be invited to complete 30 consecutive days of daily EMA-SHAPS entries by using the cross-device platform.

### Eligibility Criteria

#### Inclusion Criteria

Inclusion criteria (both cohorts) are as follows: adults aged ≥18 years with sufficient capacity to provide informed consent, ability to communicate in Chinese, regular access to a smartphone or web-enabled device, and willingness to spend approximately 15-20 minutes completing the baseline questionnaire battery. For the clinical cohort, participants must be outpatients aged 18-65 years at the collaborating psychiatry clinic with a treating psychiatrist–assigned ICD-10 mood disorder diagnosis in the F32-F39 range documented in the medical record. For the nonclinical cohort, participants must be university students aged 18-23 years recruited from campus settings.

#### Exclusion Criteria

The exclusion criteria are as follows: other severe mental disorders such as schizophrenia-spectrum disorders or bipolar disorder or neurological/medical conditions that substantially impair cognitive function; acute high suicide risk at screening, as judged by the treating clinician; and other medical or functional reasons that would prevent the participant from using the digital platform or from sustaining repeated questionnaire completion. These exclusion criteria apply to both cohorts and are intended to ensure participant safety and the feasibility of the 30-day EMA protocol.

### Measures and Data Collection

The following measures were used:

1. SHAPS with EMA prompts (14 items, 4‑point responses) at baseline and scheduled intervals via the app.
2. Usability/face‑validity questionnaires; expert content validity indexing for platform features and visualizations.

For the clinical cohort, primary psychiatric diagnosis, comorbid conditions, and concurrent treatments (eg, psychotropic medications, psychotherapy) will be extracted from the medical record at baseline. For the nonclinical cohort, participants will complete a brief self-report form on past and current mental health treatment. These variables will be summarized descriptively to characterize the sample and to explore whether anhedonia trajectories differ by diagnostic profile or treatment exposure; no stratified hypothesis tests are planned in this pilot.

Feasibility outcomes will focus on adherence, timing fidelity, and patterns of missing data. For each participant, adherence will be defined as the proportion of completed daily EMA-SHAPS entries out of the 30 scheduled days. A priori, we consider adherence of ≥70% (≥21 of 30 d completed) and the absence of any run of ≥5 consecutive missing days as an acceptable benchmark for individual-level feasibility. Timing fidelity will be defined as the proportion of completed entries submitted on the same calendar day as the scheduled prompt, with ≥80% same-day completion regarded as an acceptable benchmark. We will summarize the distribution of these metrics and report the proportion of participants who meet the adherence and timing thresholds in the clinical and nonclinical cohorts separately.

### Digital Platform and Moving Average Indicators

The digital platform will have the following features.

1. Cross‑device capture, secure backend, dashboards for participants and clinicians
2. Visualization of SMA (7/14/30 d); candidate alert logic (fast–slow moving average crossover; deviation from individualized baselines)
3. Data quality monitoring for missingness and compliance; encryption for data in transit and at rest

**Figure 1:**
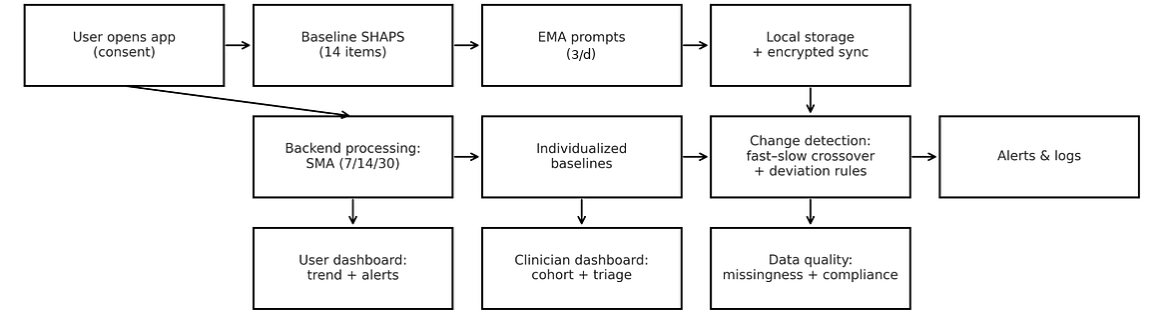
End‑to‑end user flow: app entry and baseline SHAPS → EMA capture → backend moving average computation (SMA) → change detection → alert logging → dashboards. EMA: ecological momentary assessment; SHAPS: Snaith–Hamilton Pleasure Scale; SMA: simple moving average.

**Figure 2:**
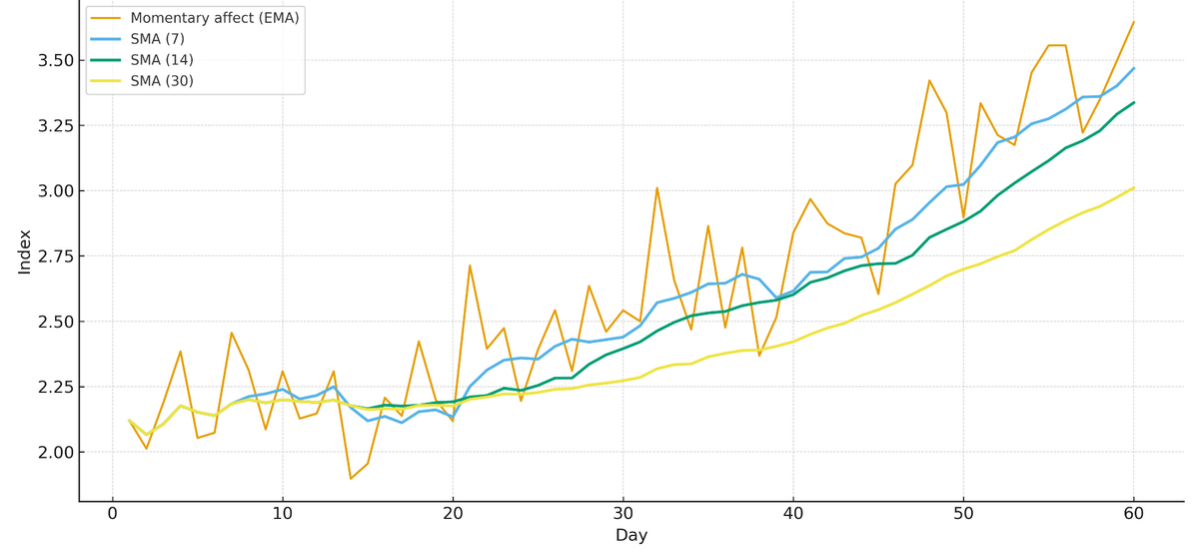
Example of a trend/alert panel for EMA-SHAPS showing daily values and simple moving averages (7/14/30 d). The 30-day SMA serves as a participant-specific monthly baseline; alert badges are generated by prespecified candidates (eg, fast–slow moving average crossovers; deviations from individualized baselines). Display is for research use in part I and does not drive clinical action. EMA: ecological momentary assessment; SHAPS: Snaith–Hamilton Pleasure Scale; SMA: simple moving average.

An example of the end‑to‑end user flow is shown in Figure 1, and the trend/alert panel is shown in Figure 2.

### Sample Size and Statistical Analysis

As a development and validation pilot, part I is designed primarily to provide feasibility, usability, and descriptive analytic information rather than to support formal hypothesis testing. We will recruit approximately 24 participants (≈12 per cohort), consistent with a widely cited “12-per-group” rule of thumb for pilot and feasibility studies that aim to estimate variability rather than detect small effects [26]. All primary analyses will be descriptive, reporting means, standard deviations, medians, interquartile ranges, and 95% CIs, where informative.

For each participant and each time window, 7-, 14-, and 30-day SMAs of EMA-SHAPS scores will be computed only when at least 50% of days in the window contain valid entries (ie, ≥4 of 7, ≥7 of 14, and ≥15 of 30 d, respectively). Windows that do not meet this criterion will be flagged as data sparse and excluded from primary SMA summaries. We will distinguish intermittent missingness (isolated missing days or gaps of <5 consecutive days) from systematic missingness (gaps of ≥5 consecutive missing days or early dropout). Primary analyses will not impute missing values; instead, we will conduct sensitivity analyses that (1) compare SMA summaries with and without windows spanning systematic missingness and (2) examine how adherence levels relate to the stability and interpretability of SMA-based indicators. Internal consistency of EMA-SHAPS will be summarized using Cronbach α, and exploratory correlations between daily scores and SMA windows will be reported with appropriate caution, given the limited sample size.

### Ethical Considerations

This study protocol was approved by the Institutional Review Board of Tsaotun Psychiatric Center, Ministry of Health and Welfare, Taiwan (approval 114006). All participants will provide written informed consent before any study procedures. During consent, participants are informed that participation is voluntary, that they may decline to answer any question, and that they may withdraw from the study at any time without any consequences for their clinical care or academic standing. The collected data will be pseudonymized so that no personally identifiable information is included, and only the research team will have access to the analytic data set. All data will be stored securely on password-protected servers for at least 7 years after study completion. As a token of appreciation, participants in each cohort will receive a gift voucher valued at NT $100 (approximately US $3) upon completion of the 30-day EMA-SHAPS assessments and end-of-pilot questionnaires.

## Results

Recruitment for the year-1 feasibility pilot will begin following institutional review board approval (approval date: June 10, 2025) and completion of platform qualification, with data collection scheduled from March 2026 through May 2026. No data have yet been collected or analyzed. Enrollment will proceed in parallel at the clinical and nonclinical sites until the predefined target sample is reached. Because each participant contributes a 30-day EMA-SHAPS series, the last participant is expected to complete end-of-pilot assessments approximately 30 days after enrollment closure. Data analyses will then be performed and reported. A month-by-month timeline of the planned milestones is provided in Table 2.

In this year-1 feasibility pilot, we also expect to produce a working cross-device prototype that delivers daily EMA-SHAPS assessments and visualizes 7-, 14-, and 30-day SMA trends at the individual level. We will obtain expert-rated content validity indices for key interface elements and alert explanations, together with participant-reported usability and interpretability metrics (eg, System Usability Scale scores). In addition, we will generate feasibility metrics for EMA completion, patterns of missingness, and timing fidelity, as well as a descriptive characterization of moving average–based indicators (eg, crossovers, deviations from individualized baselines, and gaps in data coverage) and preliminary parameter ranges that can be carried forward to refine the design and powering of part II.

## Discussion

### Principal Anticipated Findings

Year 1 is expected to deliver a functioning cross-device platform that supports daily EMA-SHAPS data collection and visualizes 7/14/30-day SMA trends at the individual level. If recruitment and adherence targets are met, the pilot will provide (1) feasibility metrics for daily anhedonia monitoring across clinical and nonclinical cohorts, (2) usability and perceived interpretability indices for the user interface and trend/alert panels, (3) expert content-validity ratings for key visual elements and explanations, and (4) preliminary descriptive information on the behavior of SMA-based indicators under real-world missingness patterns. These outputs are intended to inform refinement of platform features and alert parameters and to guide the design and powering of a subsequent controlled evaluation rather than to establish clinical efficacy on their own.

### Comparison With Prior Work

Existing digital mental health platforms increasingly use smartphones and wearables to capture intensive longitudinal symptom data and ecological context [8-11,23,24]. However, most current anhedonia assessments still rely on cross-sectional questionnaires or infrequent follow-ups and do not systematically integrate early-warning indicators derived from time-series analysis or moving average theory [12-18]. By focusing on EMA-SHAPS and SMAs, this protocol adapts tools that have been widely used in engineering and epidemiologic surveillance [15-18,20-22] to a clinically meaningful affective dimension. Rather than proposing complex forecasting models, we prioritize transparent, user-friendly visualizations (eg, short/long SMA crossovers, deviations from individualized baselines) that can be explained to patients and clinicians and empirically calibrated in later phases.

For anhedonia specifically, prior work has validated SHAPS cutoff scores for between-person discrimination [4-7], but there is little guidance on how within-person fluctuations should be summarized or visualized for ongoing self-management. This pilot responds to that gap by testing whether an SMA-based dashboard is acceptable and interpretable to both end users and experts while keeping analytic choices simple enough to be incorporated into routine care if later evaluations support benefit.

### Strengths and Limitations

The proposed study has several strengths. It uses a cross-device platform that mirrors real-world technology use, includes both clinical and nonclinical cohorts to sample a range of anhedonia severity and treatment contexts, and prespecifies adherence thresholds and missing-data rules for computing SMA indicators. The protocol also combines quantitative feasibility metrics with expert content-validity ratings, providing converging information about whether the platform is usable and conceptually sound.

At the same time, key limitations reflect the pilot nature of part I. The planned sample size (~24 participants) is sufficient for feasibility and usability assessment but underpowered for robust inferential tests of trend stability, variance components, or alert performance. All analytic checks of SMA behavior must therefore be interpreted as preliminary and descriptive. In addition, recruitment from a single outpatient clinic and 1 university limits generalizability, and we will not examine clinical outcome changes or treatment decisions in this phase. Anticipated practical challenges include sustaining EMA engagement over 30 days, handling missing and noisy data, managing cross-device heterogeneity (eg, operating systems, screen sizes), and calibrating individualized alert thresholds without overtriggering. To partially mitigate these challenges, we prespecify adherence and timing benchmarks, implement in-app reminders and data-quality monitoring, standardize onboarding and support procedures, and treat all alert parameters as modifiable candidates for later evaluation rather than fixed clinical rules.

### Future Directions

If the pilot demonstrates acceptable feasibility, usability, and content validity, subsequent work (part II) will focus on evaluating the analytic and clinical performance of moving average–based indicators in larger and more diverse samples. This may include testing whether specific SMA-derived features (eg, crossing events, sustained deviations from baseline) predict movement toward or away from validated SHAPS cutoff points or broader depressive symptom outcomes, and whether providing dashboard feedback to patients and clinicians improves shared understanding of anhedonia trajectories. Over time, the platform could be extended to incorporate additional EMA items, passive sensor data, or adaptive sampling schedules, provided that the resulting complexity remains interpretable for end users.

### Dissemination Plan

In line with the emphasis on transparent and reusable digital health research, we plan to disseminate findings from this methodological pilot through peer-reviewed publications and conference presentations in psychiatry, nursing, and digital mental health. Subject to institutional and funder policies, we will also consider sharing nonidentifiable platform specifications, example pseudonymized dashboards, and analytic codes for SMA-based indicators via institutional repositories or open-source platforms. These dissemination activities aim to support replication, integration into related research programs, and eventual translation into routine mental health care if later evaluations demonstrate clinical utility.

### Conclusion

Combining EMA-SHAPS with SMA-based trend visualizations may provide an interpretable way to summarize daily anhedonia signals and to generate candidate early-warning indicators for clinical and self-management use. This year-1 methodological pilot is not intended to establish clinical efficacy; rather, it will deliver feasibility, usability, content-validity evidence, and preliminary analytic parameters for 7/14/30-day SMA-based indicators. These outputs will be used to refine platform features and alert logic and to support the design and powering of a subsequent controlled evaluation of the platform’s clinical utility.

## Appendix

Multimedia Appendix 1 Peer-review report from the National Science and Technology Council (NSTC), Taiwan.

## Abbreviations

EMA: ecological momentary assessment
ICD-10: International Classification of Diseases, 10th Revision
SHAPS: Snaith–Hamilton Pleasure Scale
SMA: simple moving average
