# Supplementary material for: Development and Validation of a Cross-Device Platform for Anhedonia Trend Visualization by Using Ecological Momentary Assessment and Moving Averages (Part I): Protocol for a Methodological Pilot Study
Source: JMIR Res Protoc. 2025 Dec 18;14:e84024. doi: 10.2196/84024 (PMC12757707; doi:10.2196/84024)
Supplement: Multimedia Appendix 1 [file resprot_v14i1e84024_app1.pdf]

Certificate of Government Peer Review & Grant Approval

National Science and Technology Council (NSTC), Taiwan (R.O.C.)

Project Identification

Applicant (PI)	Yen-Chung Ho
Affiliation	Department of Nursing, Asia University, Taichung, Taiwan
Project Title	Development and Application of a Personalized Dynamic Monitoring System for Anhedonia Based on Moving Average Theory and Ecological Momentary Assessment: A New Direction in Precision and Preventive Psychiatry
Funding Agency	National Science and Technology Council (NSTC), Taiwan (R.O.C.)
Administering Institution	Department of Nursing, Asia University
Project Period	2025-08-01 to 2026-07-31
Project Number	114-2314-B-468-006-

Peer-Review Comments

Reviewer 1

The project proposes to develop an online platform to conduct dynamic assessments of anhedonia via the SHAPS, applying Moving Average (MA) theory to monitor depression, evaluate the predictive validity of an optimized SHAPS cutoff, and implement early-warning detection.

1) Novelty & Significance: Using SHAPS to assess depression, the provided data show unsatisfactory ROC performance. For the nonclinical group, the basis for depression classification is not clearly described, making it hard to verify the fundamental premise of the study.

2) PI Performance: Sufficient publication record.

3) Weaknesses & Specific Suggestions:

1. The ROC performance does not demonstrate that SHAPS can discriminate depression.

2. Longitudinal repeated administration of the same questionnaire for clinical follow-up may be impractical; patients may lack motivation to repeatedly complete such scales.

3. Why not measure depression directly? Substituting SHAPS for a depression scale lacks a pragmatic rationale.

4. The platform lacks basic description and specification.

5. An early-warning mechanism is mentioned, but standards/targets and methodological details are not specified; terms such as platform, innovation, and early warning are repeated without clear explanations.

6. MA theory is generally meaningful with large datasets. The study proposes only 12 participants and does not specify whether they are clinical patients, campus recruits, or university students.

4) Summary: The study aims to warn of depression, but the target of warning (severity vs behaviors) is not defined. If questionnaires are used, why not use a depression scale directly? Substituting with SHAPS entails a gap, reflected in the ROC performance. Applying MA theory with a very small sample reduces the study's value.

Reviewer 2

The study aims to build a MA-based digital platform for dynamic monitoring of anhedonia and, via this platform, to: verify the feasibility of applying MA theory to mental health assessment; build an early-warning model for nonclinical individuals; establish a relapse-monitoring model for clinical depression cases; and test whether the MA-based platform outperforms traditional anhedonia assessment.

1) Novelty & Significance: Knowledge gap: Most apps target depression/anxiety, with fewer focusing on anhedonia which may serve as an early indicator for depression. There is currently no individual-level SHAPS cutoff and no dynamic monitoring tool specifically for anhedonia. Applying MA theory—traditionally used in economics/finance and enabled by big data—to emotional time series is novel and may reduce misclassification due to noise, per the applicant's feasibility analysis.

2) PI Performance: 1) Three SCI first-author papers; two coauthored papers; recent presentations at international conferences. 2) Prior preliminary research on anhedonia assessment tools provides relevant background. 3) Co-investigator on an NSTC project regarding AR/VR-based adolescent tobacco prevention (114-2410-H-468-001-; from 2025/01/01); no prior NSTC final reports/outcomes yet.

3) Weaknesses & Specific Suggestions:

1. Is SHAPS the only instrument for assessing anhedonia? Consider whether physiological, psychological, or social parameters/signals can also be incorporated.

2. The experimental approach is sketched but needs more detailed operationalization (e.g., RCT randomization procedures; whether blinding is applied).

3. While MA can mitigate noise-driven misjudgment, it may be insensitive to short-term changes; both aspects should be addressed.

4. For the clinical cohort (18–65), consider differences in needs and acceptance of digital interventions across age groups.

5. Given reliance on SHAPS, add referent indicators/outcomes.

4) Summary: Building an MA-driven anhedonia platform is innovative, but theoretical and literature support should be strengthened—ideally with a small pilot. Enrich the literature on anhedonia and explore additional parameters/signals beyond SHAPS. More comprehensively map the domestic and international evidence base.

Reviewer 3

The PI plans to build an anhedonia monitoring EMA platform grounded in MA theory and evaluate its performance with a clinical depression population and a nonclinical university student population.

1) Novelty & Significance: The topic is not academically novel but has clinical innovativeness and importance in Taiwan.

2) PI Performance: Overall above-average scholarly performance; good publication quality; performance above the peer average.

3) Weaknesses & Specific Suggestions:

1. No prior results in digital mental health platform development; outsourcing introduces execution risk. Recommend concentrating pilot activities: complete platform development and early data collection in Year 1 to yield preliminary outcomes for feasibility assessment.

2. The PI lacks prior experience with EMA and with combining MA and EMA; the team's experience in these areas is not described, reducing persuasiveness. More thorough planning is needed.

3. The plan's design and write-up lack novelty and uniqueness and are somewhat incomplete.

4) Summary: As a new faculty member, the PI is advised to evaluate Year-1 performance before continuing.

Reviewer 4

The project aims to develop an MA+EMA anhedonia monitoring digital platform and validate its performance in clinical (depression) and nonclinical (university student) groups.

The integrated application has potential value, but the theoretical basis should be further strengthened.

1) Novelty & Significance: Publications are above average among peers, but the PI lacks experience in developing digital mental health platforms and has no prior MA or EMA research background.

2) PI Performance: —

3) Weaknesses & Specific Suggestions:

1. Outsourcing and postponing data collection to Year 2 create risk; recommend integrating data collection earlier to ensure feasibility.

2. Add EMA and MA experts to the team to ensure methodological rigor.

4) Summary: Recommendations: (1) Expand the sample size and start SHAPS data collection earlier to ensure feasibility; (2) Broaden the team and strengthen EMA/MA expertise; (3) Describe data processing details to improve transparency and reproducibility; (4) Consider strategies to enhance the platform's application value.
